# Neonatal Suckling, Oxytocin, and Early Infant Attachment to the Mother

**DOI:** 10.3389/fendo.2020.612651

**Published:** 2021-02-08

**Authors:** Raymond Nowak, Frédéric Lévy, Elodie Chaillou, Fabien Cornilleau, Juliette Cognié, Pierre-Guy Marnet, Peter D. Williams, Matthieu Keller

**Affiliations:** ^1^ Unité de Physiologie de la Reproduction et des Comportements (PRC), INRAE, CNRS, IFCE, Université de Tours, Nouzilly, France; ^2^ Département Productions Animales, Agroalimentaire, Nutrition (P3AN), Agrocampus Ouest, Rennes, France; ^3^ Department of Medicinal Chemistry, Merck, West Point, NY, United States

**Keywords:** attachment, oxytocin, newborn, suckling, sheep, infant bond formation, social behavior

## Abstract

The neuropeptide oxytocin (OT) promotes maternal care and social affiliation in adults but its importance in infant attachment still remains unknown. True animal models of infant attachment are extremely rare, and the sheep (in complement to non-human primates) is one of the few that provides the opportunity to investigate its neuroendocrinological basis. In the lamb, access to the udder has strong rewarding properties for the establishment of a preferential relationship with the mother. Therefore, the present study explored the possible involvement of OT through its release during close social contacts with the mother. The first experiment revealed that lambs having free access to the udder from birth onward developed, by 12 h of age, a clear preference for their mothers over another maternal ewe. Delaying access to the udder for six, four or even only 2 h starting at birth, by covering the ewe’s udder, resulted in the lack of such a preference without affecting general activity. These effects persisted in most cases at 24 h but by 72 h of age a bond with the mother was clearly expressed. Experiment two showed that social interactions with the mother were followed by a release of OT in the plasma when lambs had the possibility to suckle. Non-nutritive interactions were without effects. Preliminary data on two subjects suggested that OT might also increase in the cerebrospinal fluid after suckling. Finally, in the third experiment, oral administration of a non-peptide OT receptor antagonist (L-368-899, Merck) over the first 4 h after birth led to decreased exploration of the mother’s body compared to lambs receiving saline, and impaired the expression of a preference for the mother at 24 h. The effects were no longer observed at 48 h. Our findings demonstrate that both delayed access to the mother’s udder and OT receptor antagonist alter the onset of mother preference in newborn lambs. This suggests that central OT facilitates the development of filial attachment through its release during suckling.

## Introduction

Preferential interactions and mutual bonding in mammals are usually linked to litter size, mobility of the infant, duration of parental care and sociality (1–4). Bonds are more likely to emerge in species raising only one or two young, highly mobile, and where the risk of misdirecting maternal care is high. Mothers recognise their young on individual features and develop selective care (5–7). Reciprocally, the newborn acquires a multisensory image of the mother that forges its behavior in a preferential manner towards her (8). This relationship may give rise to an attachment bond which refers to an affectional tie that one individual forms to another. Elaborated in humans first (9), this concept was then extended to other mammalian species (1).

The onset of parental and infant behaviors relies on the involvement of specific proximate factors: neurotransmitters-neuromodulators acting during the perinatal period, and learning processes of sensory information emanating from the two partners. Extensive research over the last decades has greatly improved our knowledge of the neuroendocrinological and neurobiological bases of parental care, infant behavior and social affiliation. While proven valuable to understand how proximate factors control the onset or the expression of such behaviors, two observations can be made from the available literature. The first is that we have a far better understanding of the mother than of the infant [e.g., (10, 11)]. The second is that studies focusing on infant behavior have favoured altricial species (12). As an international experimental model in neurobehavioral science, the rat pup was used to investigate early olfactory learning (13, 14), social play (15), maternal deprivation (16), and infant attachment (17, 18). The importance of these studies in understanding mother-young interactions is unquestionable but relates specifically to precocial species. A direct parallel between rat pups’ early learning abilities and the development of infant attachment is not conceivable, since until now there is no evidence that they develop an affectional tie with a specific caregiver as defined by Ainsworth (9) in humans and Gubernick (1) in non-human species. Therefore, it appears that the biological mechanisms leading to the development of infant attachment are still vastly unknown.

Infant attachment can be inferred from behavioral and neuroendocrinological responses: preference for the attachment figure (the caregiver) over another, maintenance of proximity, and distress responses following involuntary separation (increased vocalization, activation of the hypothalamic-pituitary-adrenal axis) are the most commonly used indices (1, 19). Clear experimental evidence comes from only a few species: some non-human primates [rhesus monkey: (20); titi monkey: (21, 22)], the guinea pig (19) and the sheep (2). Except for rare studies in rhesus monkeys (23, 24), our knowledge of the internal factors leading to the development of infant attachment is based on work carried out in sheep (25). Sheep are precocial mammals and their young develop a strong and enduring social bond with the mother that can be truly qualified as attachment (26–28). The initial step is the development of a preference for the mother observable in most lambs within 12–24 h after birth (29). The development of such preference is a learning process that relies on neuroendocrinological changes based on the success of the first suckling episodes. Should access to the udder be prevented for a few hours, lambs show a delay in discriminating their mothers from other maternal ewes (30). It appears that ingesting colostrum in the hours after birth is a key factor for the development of a preference for the mother (31, 32). The neonatal suckling activity triggers the release of cholecystokinin (CCK) in the blood, a gastrointestinal hormone which activates vagal afferent fibres and the brain stem (33, 34). Blocking the cholecystokininergic system with a specific antagonist that binds to peripheral receptors at birth prevents the development of a preference for the mother (35–37). While the initial internal factors involved in the onset of the bond seems to be located in the gastrointestinal tract, contributions of brain substrates are unclear. All we know is that similar outcomes are obtained with the use the opioid receptor antagonist naltrexone, revealing that the opioidergic system is involved (38).

Massive accumulation of knowledge concerning the involvement of oxytocin (OT) in social affiliation and bonding raises the question as to what extend this neurohormone may also be involved in the initial steps leading to infant attachment. Yet, among the numerous reviews focusing on OT, only a few refer to the infant (39–41). Unlike the adult, the regulation of infant social bonds has received less attention probably because being small, immature and fragile, there is a true difficulty to intervene and collect neuroendocrinological information. We know however, that in humans OT plasma levels are higher in the neonate than in the mother, they decrease after birth, and are higher in infants born by vaginal delivery than in those born by caesarean section (40, 42). In a situation where parents were asked to engage in “play-and-touch” with their 4–6 month-old infants, Feldman et al. (43) have shown that salivary OT increases both in parents and children following contact interactions, and that OT concentrations are linked to the amount of social engagement. In 7- to 11-day-old calves, Lupoli et al. (44) have shown that suckling at the udder triggers a rise of OT that is much higher than drinking milk from a bucket, even in the presence of the mother. This suggests that sensory stimulations during mother-young interactions potentiate the neuroendocrine response. Finally, it has been shown that the oxytocinergic system of the hypothalamus is activated in artificially fed lambs interacting with their human caregiver, with whom they express affiliative behaviors (45). The oxytocinergic system is obviously active and reacts to social/somatosensory stimulation in infant mammals that are known to develop a bond with their mothers.

In the light of the above results, we examined in the lamb the links between: (i) early suckling activity and the development of a preference for the mother, (ii) social interactions and OT release, and (iii) OT and early bonding process. To achieve our goal, we ran three experiments. In the first, we followed the spontaneous activity of the lamb during the 6 h after birth, focusing on the suckling activity. We also explored to what extend a delay in successful suckling could affect the initial expression of a preference for the mother. We hypothesized that the magnitude of the consequences would be linked to the duration of the delay. In the second experiment, we followed plasma OT levels when lambs engaged in close interactions with their mothers. We hypothesized that OT would be released during social encounters, and suckling would potentiate the response. An attempt was also made in a preliminary trial to measure OT in the cerebrospinal fluid. In the third experiment, we examined the hypothesis that blocking the OT system at birth with a specific receptor antagonist (L-368,899) would alter the development of a preference for the mother.

## Materials and Methods

### Subjects

We used multiparous Ile-de-France ewes and lambs from the “Unité Expérimentale de Physiologie Animale de l’Orfrasière” (INRAE Val de Loire, France; https://doi.org/10.15454/1.5573896321728955E12). Pregnant ewes were kept indoors as a single flock and fed hay and pellets of concentrate according to their requirements. Water was provided at libitum. Two weeks before the due lambing date, they were taken to an experimental building and housed in individual pens (2 m x 1 m). Social interactions between neighbours remained possible through the upper part of the metal hurdles. During this time, they were accustomed to human voice and presence by being gently stroked and hand-fed one by one on a daily basis. When birth was imminent, an experimenter assisted each ewe in the last stage of expulsion. The presence of a second foetus was checked manually and, should this be the case it was pulled out to standardize time of birth within a litter. In case of triplets, the lighter lamb was removed and fed with artificial milk meaning that lambs were reared as litters of twins at the most. This was decided to make sure that each lamb had access to a teat and minimized competition at the udder. Matching the time of birth was required for experiments 1 and 3 due to the treatments imposed on lambs that needed to be synchronized in twins (see below). Lambs were ear-tagged and weighed, and returned to their home pen within minutes after birth. Human interference had no visible effect on subsequent ewes’ maternal behavior.

### Experiment 1. Postponing Suckling at Birth Alters the Onset of Early Infant Attachment

#### Suckling-Deprivation Method

Subjects were 30 ewes and their 54 lambs. Lambing spread over a period of five days and lambs were randomly allocated into one of the following groups, taking gender and litter size into account. Non-deprived subjects had free access to the mother’s udder from birth onwards (Control group: n=16; six singles and 10 multiples, nine females and seven males). In the other groups, lambs were denied of suckling by covering the udder immediately after parturition for 2 h (0–2 h: n=14; four singles and 10 multiples, nine females and seven males), 4 h (0–4 h: n=14; seven singles and seven multiples, nine females and five males), or 6 h (0–6 h: n=10; three singles and seven multiples, seven females and six males). The cover was made of thin, elastic and silky material to allow maternal warmth and body odours to be perceived by neonates. Once the cover was removed, lambs were guided to the teats until they could ingest colostrum unaided. This procedure ensured that suckling deprivation would not extend beyond the set period of 2 h, 4 h, or 6 h. To standardize animal handling, lambs from the Control group were also guided to their mothers’ udder 6 h after birth. Ewes and lambs were taken to two nearby adjacent rooms (10 x 8 m) 6 h after parturition and reared in groups of 12 mothers and their young; previous studies had shown that living with other partners facilitated the development of a bond with the mother (46).

#### Behavior, Temperature, and Body Weight

Spontaneous neonatal behavior was recorded in the Control group only by direct observation from birth until 6 h later (5 min every 20 min). Latency to stand up (time between birth and first standing for at least 10 s), latency to find the teat (time between birth and grasping the teat for at least 10 s) and suckling duration were noted. This was done in order to have precise timing of neonatal suckling activity in non-deprived subjects, and make sure that access to the udder was successfully achieved before lambs from the three other groups. In addition, the number of bleats emitted, the times spent standing and exploring the maternal body (lamb’s nuzzle in contact with the skin/wool of the ewe) were also taken into account. Lambs were weighed at birth and then at 6 h, 12 h, 24 h, and 48 h of age, and rectal temperature was taken using a digital probe thermometer (range: 32.00 ~ 43.00°C, accuracy: ± 0.10°C).

#### Testing Apparatus

The testing pen was located in a room away from the two flocks (approximately 25 m). It consisted of 1-m-high metal barriers delimiting a triangular area (5 × 4 × 4 m) covered with straw (for detailed description see: 31, 37). The longer side of the triangle was made of three pens side by side. Two of them were designed to restrain the mothers (stimulus pens: 2 x 1 m) while the smaller middle pen (1 × 1 m) separated them. In the opposite corner was the starting pen for the lambs (0.7 × 0.7 m). A 50-cm wide area in front of the stimulus pens was considered to be a contact zone. Once in it, close physical interactions between lambs and ewes were possible since the bars of the pens left sufficient space to allow reciprocal nosing and sniffing. A lamb was considered to be in a specific zone once its forelegs had crossed the limit of that zone.

#### Two-Choice Test Procedure

All the lambs were repeatedly tested at 12 h, 24 h, and 72 h after birth. The mother and an unfamiliar mother were brought and placed in either of the stimulus pens. Because lambing spread over a few days, it was possible to select two ewes that had given birth approximately at the same time. The lamb to be tested was placed in the starting pen, standing, with its head towards the ewes. The experimenter triggered the video recorder and left the room. In litters of twins, both lambs were tested one after the other but the location of the ewes in the stimulus pens was reversed. Care was taken so that left and right sides occupied by mothers were evenly distributed in each group and age class. In addition, their position in the three consecutive tests was systematically reversed so that lambs did not associate a specific place with the presence of their mothers, which could influence subsequent choices (47). Once the testing session was finished, ewes and lambs were reunited and returned to their rearing room. The test lasted 5 min when lambs were 12 and 24 h old. At 72 h of age, the duration was limited to 3 min since as lambs grow older they are more inclined to walk away from their mothers after making initial contact and explore the surroundings (29). For each test, the latency to reach a contact zone, and the total time spent in them was recorded. The preference for the mother was assessed in two ways. Firstly, the times spent in the contact zone near the mother and the unfamiliar ewe were compared. Secondly, for each subject, an index of preference (IP) was calculated as follows: (time spent near the mother minus time spent near the alien ewe) divided by (total time spent near either ewe). This index expressed the ability of the lamb to choose between the two ewes and ranged between two extreme values. The maximum was 1 when a lamb spent all of the time near its mother, and the minimum was -1 should it spent all of the time near the unfamiliar ewe. A lamb was considered to display a clear preference for its mother if IP ≥ 0.33, a clear preference for the unfamiliar ewe if IP ≤ -0.33, and no preference in the other cases. The threshold (± 0.33) delimiting the three classes of lambs was defined according to the results obtained in previous study (31, 36). Only lambs spending more than 1 min in the contact zones were taken into consideration in the analysis of IP values.

### Experiment 2. Suckling Triggers the Release of OT in Lambs

#### Blood Sample Collection

Seven female lambs reared in individual pens (2 x 1 m) with their mothers were used in this experiment. They were accustomed to human presence from the day of birth since providing straw, hay and grains reinforced positive human-animal relationships. Lambs were 2 weeks old at the beginning of the experiment, an age that was chosen on three criteria. Firstly, performing serial blood sampling on neonates is difficult due to their small size. Secondly, serial blood sampling requires human interventions and both ewes and lambs need to be trained to get used to them. Thirdly, as lambs grow older their natural suckling frequency declines (48) and they can easily spend several hours without needing to be fed. In conditions where lambs are artificially fed, drinking milk from a bucket fitted with rubber teats, only two to three meals per day are provided (49). Our methodology, which required a period of over-night fasting, was then suitable. One week before the experiment started, lambs were separated from their mothers for 2–3 h per day with wire mesh netting that divided the home pen into two areas. The fact that lambs could see, hear and smell their mothers and that only two familiar experimenters collected the blood samples, minimized stress responses.

Blood samples were taken from the left jugular vein by fully trained personal using vacutainers. The trial was made of three sessions, each separated by a two-day-long resting period. The night before each session (08:00 p.m.), lambs were separated from their mothers with wire mesh netting. On session 1, lambs were reunited with their mothers and were allowed to make contact and suckle for 3 min after which they were separated again. On session 2, lambs were reunited with their mothers for 3 min but a cover, like in Experiment 1, prevented access to the udder. Session 3 repeated session 1, testing whether the OT response would improve due to lambs habituating to the complete procedure. In all three sessions, blood samples were taken 30 min before lambs were reunited with the mother, just before reunion, after 3 min of nutritive or non-nutritive interaction with the mother, and then 5, 10, and 15 min after the end of the contact period. Five millilitres of blood were collected for each sample in heparinized tubes kept on ice. All blood samples were taken in the home pen within 10-30 s after the experimenters restrained the lamb. After centrifugation at 4000 rpm at 4°C, plasma was stored at -20°C until assayed. All blood samples were taken in the morning between 08:00 and 11:00 a.m. OT levels were determined by enzyme immunoassay using acetylcholinesterase conjugate and second antibody coated microtiter plates as solid phase, together with prior extraction to improve sensitivity and specificity by reducing cross-reaction with bound OT to neurophysin (50). The limit of detection was 1.5 pg/ml^-1^, and the inter- and intra-assay coefficients of variation were 13 and 8.6% for 4 pg/ml^-1^, respectively. The cross reactivity was low with all known OT-like molecules and constitutive peptide sequences (< 0.4%) except with the Tyr-Pro-Leu-Gly-NH tripeptide tail (12.5%).

#### Neurosurgery

Neurosurgery had never been performed on infant lambs before and this work is a preliminary report. Only two male lambs were used to adapt the procedure developed in adult sheep (51, 52). Such procedure required excluding the mothers from the protocol as they could interfere with the wound and cause infection. Therefore, lambs were separated from their mothers the day after birth and reared together with artificial milk in straw-bedded pens (4 m x 2 m). At two weeks of age, they were transported to the surgery room, anesthetized with an intravenous injection of Clorketam 1000^®^ (ketamine 2–7 mg/kg) and Rompun 2%^®^ (xylazine 0.05 mg/kg) followed by close-circuit halothane (2.5%–3%). A catheter was inserted into their left jugular vein in order to perfuse lidocaine (20 µg/kg/h), ketamine (0.3 mg/kg/h), and morphine (0.05 mg/kg/h) while a blanket covered them on the surgery table. Subjects were placed in a stereotaxic frame adapted for sheep. After skin incision, the surface of the skull was exposed and a stainless steel cannula (18 gauge, with Luer fitting, shaft = 10 mm in length, Thiebaud Biomedical Instruments, Thonon-les-Bains, France) was inserted into the right lateral ventricle 1 cm postero-lateral to bregma. Proper position of the cannula was determined following the atlas by Richard (53) and checked by X-ray after infusion of a radio-opaque fluid (Lopamiron, Schering, France). The stainless steel cannula was secured to the bone with acrylic cement closed with obturators. A Teflon ring anchored to the skull with stainless steel screws and acrylic cement protected it. After stitching the skin, the wound was sprayed with Orospray^®^ and Aluspray^®^ two agents with antibacterial properties, and lambs received an additional intravenous injection of morphine (0.5 mg/kg). Once awake, they were given an intramuscular injection of nonsteroidal anti-inflammatory drug (Finadyne^®^, 2 mg/kg flunixin meglumin). Lambs were watched carefully over the five consecutive days during which they were treated with antibiotics (Clamoxyl^®^, amoxicillin, 7 mg/kg). Only then, did they go back to their rearing pen (2 x 2 m) and a companion lamb was added to stimulate social interactions. The two experimental lambs recovered rapidly under veterinary supervision.

#### Cerebrospinal Fluid Collection

The lambs were looked after by two trained experimenters who provided all the care, bottle fed them four times per day, and habituated them to human presence and handling. Two weeks were necessary before the two post-operative lambs were fit for CSF collection. During feeding times, these two lambs were restrained from walking by putting them in a small harness, the four legs slightly above ground. On the day of CSF collection, one experimenter was in charge of the samples while the other restrained and bottle-fed the lamb in the harness. A silastic brand catheter (Dow Corning Corp., Midland, MI, USA) was inserted through the ventricular cannula. The other end was linked to a peristaltic pump (Minipuls 2, Gilson, Villiers-le-bel, France) *via* PVC tubing, and CSF could be extracted at a specific rate (30 µl/min). Samples were collected every 5 min to obtain 150 µl of CSF per fraction. Once the peristaltic pump was started, the first 150 µl were discarded. Then three samples of CSF were collected: 1) before the experimenter entered the pen and bottle-fed the lambs in the harness, 2) during bottle-feeding, 3) after bottle-feeding once the experimenter left the pen. Lambs were released from the harness immediately after. For each subject CSF samples were collected every second day, in the home pen, and in the presence of the other lambs. Only one lamb was sampled on a specific day. Samples were kept on ice and stored at -20°C until assayed. OT levels were determined by the same enzyme immunoassay method as for plasma (50).

### Experiment 3. Early Infant Attachment Is Delayed by an OT-Receptor Antagonist

#### Pharmacological Treatment

The general rearing conditions were the same as in Experiment 1: pregnant ewes were accustomed to human presence, mothers and lambs remained in their individual pens (2 m x 1 m) for the first 6 h after parturition to receive the treatments, and then, were taken to larger pens (5 x 5 m) housing 3 to 4 mothers and their lambs. We used 31 lambs and 22 mothers, sex ratio and litter size were balanced between the experimental treatments. One group of lambs received saline (n=10; four singles and six twins, seven females and three males) while the others were given L-368,899 diluted in saline at a low (1 mg/kg; n=11; five singles and six multiples, seven females and four males) or high concentration (10 mg/kg; n=11; five singles and six multiples, six females and four males). L-368,899 was chosen for the following reasons: (i) it is a non-peptide that is rapidly absorbed in the blood after oral administration and remains in it for several hours [rat and dog: (54)]; (ii) it can cross the blood-brain-barrier to a significant extent in the rhesus monkey, and reduces interest for infants in adult females (55); (iii) it affects pair-bonding in the marmoset (56); (iv) it blocks OT-induced social affiliation in the naked mole-rat (57). Within a same litter, twins received a different treatment. L-368,899 and saline were administrated orally (10 ml) through oro-gastric catheters (Vygon, France), a method that had been validated before (31, 32). At the end of each treatment, lambs received a further 10 ml bolus of bovine colostrum to rinse the dead volume remaining in the catheter. The treatment was administered three times at 2 h intervals: birth, 2 h and 4 h after birth. This time-schedule was chosen on the basis of: 1) the available data on pharmacokinetics of L-368-899^®^ half-life when administrated orally (54), and 2) results from experiment 1 showing that the behavioral consequences (see results below) were more important if suckling was delayed by at least 4 h after birth.

#### Body Weight and Behavioral Observation

To check for any potential side effects of L-368-899, each lamb was observed for 10 min before orogastric administration of the bolus at 2 and 4 h after birth, and again at 6, 8, 10 and 12 h. The following behavioral parameters were examined: 1) time standing, 2) time exploring the mother’s body, 3) time spent suckling, and 4) number of bleats emitted.

#### Two-Choice Test

At 24 and 48 h of age, lambs were tested for their ability to discriminate their mothers from another maternal ewe. The aim was to assess which dose would affect the initial expression of a preference for the mother, and to determine if the effects observed at 12 h would persist over time. The testing conditions were the same as in Experiment 1.

### Statistical Analysis

Data were analyzed using XLSTAT 2017 by Addinsoft software. All variables were checked for normality using the Shapiro-Wilk test. Only birthweight, temperature, and OT data followed normal distribution and were analyzed with repeated measures ANOVA. Behavioral data were subjected to nonparametric analysis due to a lack of normal distribution. For independent samples, the Kruskal–Wallis test was used followed by the Dun *U* -test for any pairwise comparisons to detect differences between groups for ordinal values. P-values were corrected for multiple comparison with the Bonferroni test. For dependent samples, the Wilcoxon test for pairwise comparisons was chosen. The Friedman test was used to assess the effect of time within each group. It was followed the Nemeyi test for any pairwise comparison. P values less than 0.05 were considered statistically significant, and values between 0.05 and 0.10 were considered as having a statistical tendency as sample sizes were rather small and behavior was highly variable. Some P-values are also mentioned in the text and figures despite Bonferroni correction, as our sample size limited the possibility to reach statistical significance. While Bonferroni correction is recommended for multiple comparisons, its use is controversial and may depend on the circumstances of the study (58). Data are expressed as mean and standard deviation for weight, temperature and OT, and as median, 1^st^ and 3^rd^ quartile for behavior.

## Results

### Experiment 1. Postponing Suckling at Birth Alters the Onset of early infant attachment

#### Behavior, Temperature, and Body Weight

**Figure 1:**
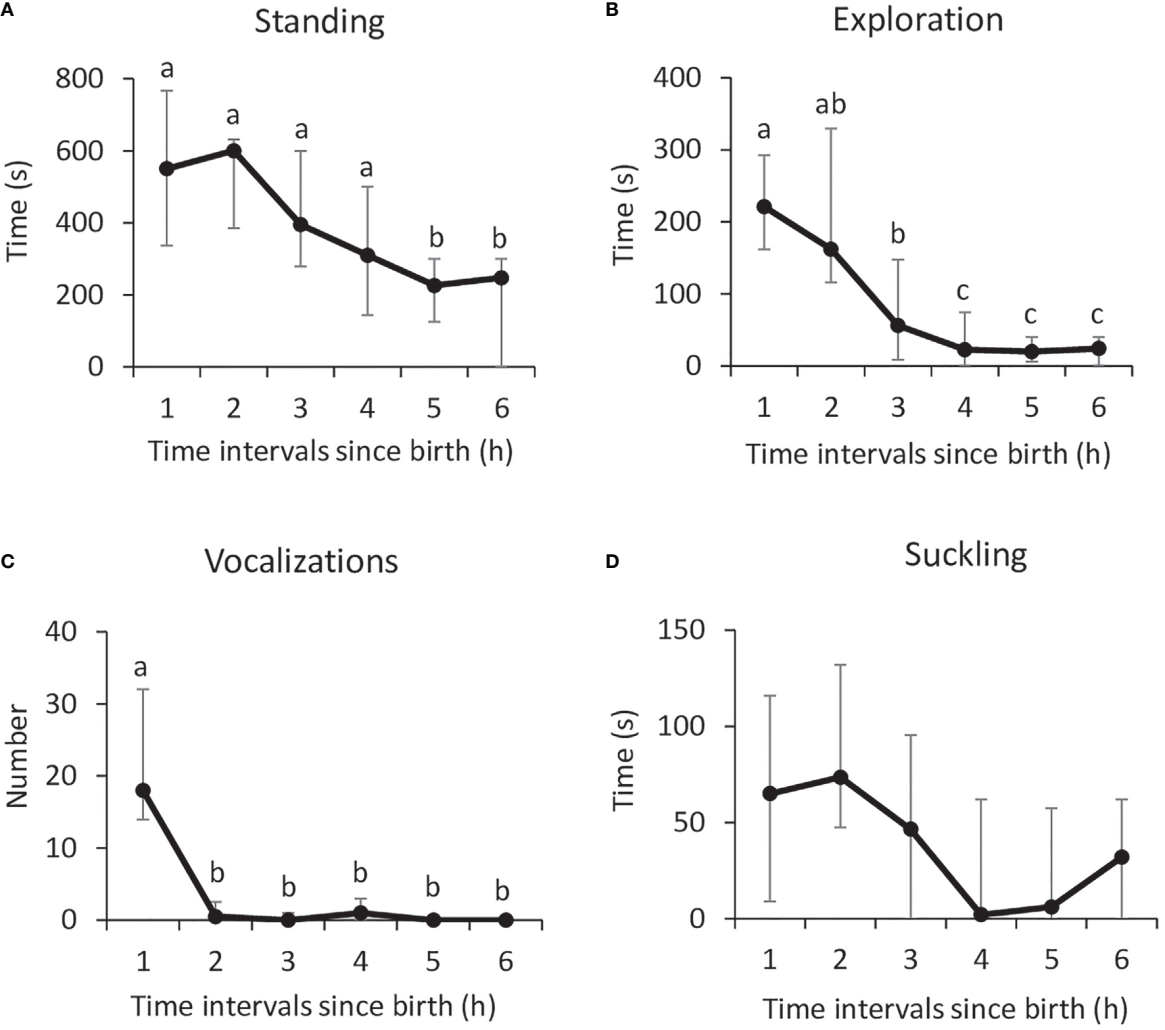
Lambs neonatal activities during the first 6 h after birth: **(A)** standing, **(B)** exploring the mother’s body (nosing, nuzzling), **(C)** number of vocalizations, and **(D)** suckling. Data (n=16) are expressed as medians and 1^st^ and 3^rd^ quartile. Data points with different letters are significantly different after Bonferroni correction (p=0.033).

Observation of the behavior in Control lambs showed that the median latency to stand up was 29.0 min [25.0–36.5] and the median latency to suckle was 55.0 min [47.7–78.0]. The time spent standing and exploring the body of the ewe, as well as the number of vocalizations, changed significantly over time (Friedman: n=16, df=5; standing: *x* ^2^ = 31.47; exploration: *x* ^2^ = 45.35; vocalizations *x* ^2^ = 48.93; p<0.0001 in all cases; **Figures 1A–C**). Lambs were most active in the first two to 3 h after birth, when trying to find the mother’s udder and then focusing on it to drink colostrum (Nemenyi post-hoc test: p<0.05 in all cases). The vocal activity was maximum in the first hour following birth, before lambs found the udder (Nemenyi post-hoc test: p<0.01 in all cases). Beyond 3 h, they rested in standing or lying position, next to their mothers. Suckling activity seemed to follow a similar pattern (Friedman, n=16, df=5; *x* ^2 =^ 48.95; p=0.027; **Figure 1D**) but pairwise comparisons did not reveal any significant difference due to the high variability.

At birth, lambs weighed from 2.2 to 5.2 kg with no difference between the four groups. Repeated measures ANOVA revealed a significant change in body weight over time (F_(3,12)_=227.437, p<0.0001) and group*time interaction (F_(12,55)_=5.939, p<0.001). Post-hoc comparisons revealed that lambs deprived of suckling for 6 h were lighter than at birth (4.1 ± 1.2 kg vs. 3.9 ± 1.2 kg, p<0.001). They were the only group of lambs losing some weight at 6 h (-0.29 ± -0.08 kg) compared to the three other groups (CTL: 0.28 ± 0.07 kg; 0–2 h: 0.45 ± 0.11 kg; 0–4 h: 0.04 ± 0.01 kg, post-hoc tests corrected with Bonferroni: p_Bon_ p<0.001). Then, the differences vanished and by 72 h of age the mean growth rate was 0.88 ± 0.27 kg. Rectal temperature followed the same trend, with a significant effect over time (F_(3,12)_=11.720, p<0.0001) but no group*time interaction. Only a tendency for a difference was observed 6 h after birth (F_(3,54)_=2.373, p=0.08), lambs deprived of suckling for 6 h having lower temperatures than Controls (39.2 ± 0.38 vs. 39.8 ± 0.32 respectively, p=0.035). However, these differences were marginal since all lambs had normal range values and none of them were considered to be hypothermic. No significant effects were found between males and females, nor between single and multiple-born lambs.

#### Two-Choice Test

When a lamb was released in the testing pen it bleated vigorously and the two ewes responded immediately. Nine lambs failed to reach the ewes at some stage during the repetitive testing sessions and were excluded from the statistical analysis: six at 12 h (one for Controls, one for 0–2 h group, two for 0–4 h group, and two for 0–6 h group) and three at 72 h (two for Controls and one for 0–6 h group). Once in the zone of contact, the other lambs tended to quiet down and explored the ewes through the bars of the hurdles while mothers kept bleating. All these lambs spent a minimum of 1 min in the contact zones (range: 91 s–279 s). No effects of gender and litter size were detected at any time.

##### Preference for the Mother at 12 h

**Figure 2:**
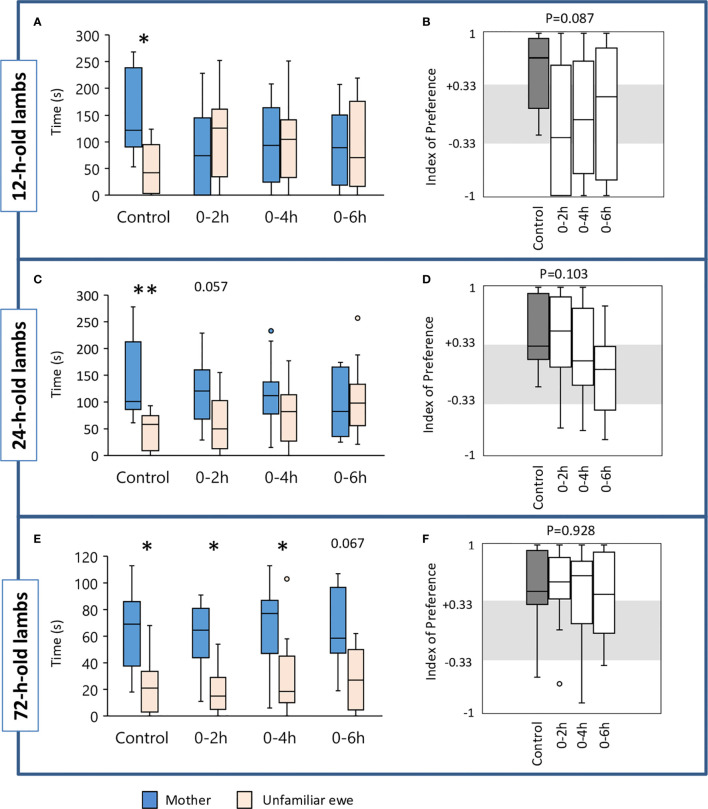
Time(s) spent in the contact zone near the mother or the unfamiliar ewe in the choice test by lambs when 12 h **(A)**, 24 h **(C)**, and 72 h old **(E)**. At birth, lambs had unlimited access to the mother’s udder (Control, n=13) or were deprived of suckling for the first 2 h (n=14), 4 h (n=14), or 6 h (n=10) after birth. Indexes of preference (IP) in 12 h **(B)**, 24 h **(D)** and 72 h old lambs **(F)**. IP>+0.33: preference for the mother; +0.33<IP<-0.33: no preference; IP<-0.33: preference for the unfamiliar ewe. Data are expresses as medians and 1^st^ and 3^rd^ quartiles. Paired box-plots associated with a star (*) are significantly different (p<0.05).

We did not find any significant difference between groups in the latency to reach the zone of contact, nor in the total time spent in the contact zones or only next to the mother or next to the unfamiliar ewe. Control lambs spent significantly more time near their mother than near the unfamiliar ewe (Wilcoxon test: V=82, n=13, p=0.012) which was not the case for any of the suckling-deprived lambs (**Figure 2A**). In addition, a tendency for a difference between groups was observed for the IP score (Kruskal-Wallis test: K_obs_=6.57, df=3, n=10 to 14, p=0.087; **Figure 2B**). Controls were the only ones having a median IP score above +0.33, meaning that more than half the lambs clearly preferred to stay next to their mothers while none displayed a preference for the unfamiliar ewe. The main feature of the suckling deprived lambs was that their choice seemed rather random since the proportion of them having an IP≥+0.33 or ≤-0.33 was rather similar (respectively: 5/14 vs. 7/14 for 0–2 h, 5/14 vs. 5/14 for 0–4 h, and 5/10 vs. 5/10 for 0–6 h). Only a few lambs did not make a clear choice. Subsequent IP pairwise comparisons using Dunn’s procedure showed that Controls (n=13) differed from 0–2 h (n=14) and 0–6 h lambs (n=10) at p=0.018 and p=0.043 respectively but this was not considered statistically significant after Bonferroni correction (set at p=0.0083).

##### Preference for the Mother at 24 h

When lambs were tested for the second time, Control and 0–2 h lambs spent significantly more time close to their mothers than to the unfamiliar ewes (Wilcoxon test: V=85, n=13, p=0.006 and V=90, n=14, p=0.020, respectively, **Figure 2C**). The other two groups failed to do so. Comparison of IP values showed a tendency for a difference between groups (p=0.103, **Figure 2D**). Control and 0–2 h lambs differed from 0–6 h lambs (p=0.030 and p=0.038). Again, this did not reach statistical significance after Bonferroni correction (set at p=0.0083). In Controls, 6/13 lambs clearly preferred their mothers (IP≥0.33), none chose the unfamiliar ewe (IP≥-0.33). In the 0–2 h group, the proportions were 7/14 and 1/14 respectively, and lambs from the 0–4 h group showed a similar trend. Only two subjects from the 0–6 h group chose their mothers. In addition, a tendency for a difference between groups was observed for the time spent near the unfamiliar ewe (Kruskal-Wallis test: K_obs_=6.30, df=3, n=10 to 14, p=0.098; **Figure 2C**). Subsequent IP pairwise comparisons using Dunn’s procedure showed that Controls differed significantly from 0–6 h lambs (n=13 and n=10 respectively, p=0.019). No significant differences were found between groups in the other variables.

##### Preference for the Mother at 72 h

When the test was repeated for a third time at 72 h of age, Controls and lambs from 0–2 h and 0–4 h groups spent significantly more time next to their mothers than next to the unfamiliar ewes (Wilcoxon test: Controls: V=80, n=13, p=0.017; 0–2 h: V=96, n=14, p=0.007; 0–4 h: V=88, n=14, p=0.028; **Figure 2E**). A trend was reached for 0–6 h lambs (Wilcoxon test: V=46, n=10, p=0.067). IP values did not differ significantly between groups (**Figure 2F**).

### Experiment 2. Suckling Induces the Release of OT in Infant Lambs

#### Peripheral Oxytocin

**Figure 3:**
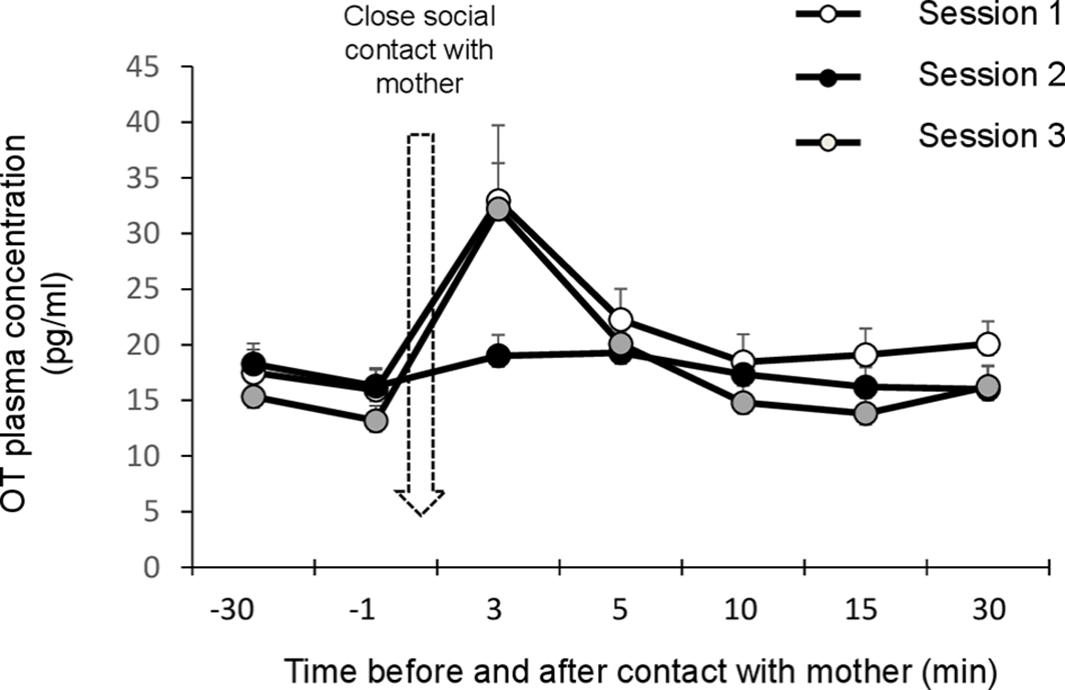
Effect a 3-min period of close social contact with the mother (arrow) on plasma oxytocin levels. Lambs (n=7) were separated from their mothers by a wire mesh before and after being reunited with the mother for 3 min. Samples were taken 30 min and 1 min before close social contact, and then 3, 5, 10, and 15 min later, in three consecutive sessions three days apart. Session 1: suckling permitted. Session 2: contact suckling prevented. Session 3: suckling permitted. Data are expressed as means and s.e.m.

When combining the sets of data points obtained before lambs were allowed to interact with their mothers, the mean OT basal concentration was 16.08 ± 0.87 pg/ml with extreme values ranging from 9.44 to 27.5 pg/ml. Repeated measures ANOVA revealed a time effect (F_(2,12)_=13.099, p<0.001) but no difference between sessions (**Figure 3**). Overall, plasma levels obtained after 3 min of close social interactions with the mother were significantly higher than at any other time (post-hoc tests corrected with Bonferroni: p_Bon_<0.001 in all cases). A significant time*session interaction was also detected (F_(6,36)_=3.106, p=0.001). In sessions 1 and 3, when access to the udder was allowed, OT plasma levels were significantly higher immediately after suckling than before (post-hoc tests corrected with Bonferroni: p_Bonf_<0.001 in both cases). In addition, in session 3 only, immediate post-suckling levels were higher than any other following time points (post-hoc tests corrected with Bonferroni: 3 min vs. 5 min, p_Bonf_=0.004; p_Bonf_ <0.001 in all other cases). No statistical differences were found between blood samples throughout session 2, when interactions with the mother excluded the possibility of suckling.

#### Central Oxytocin

**Figure 4:**
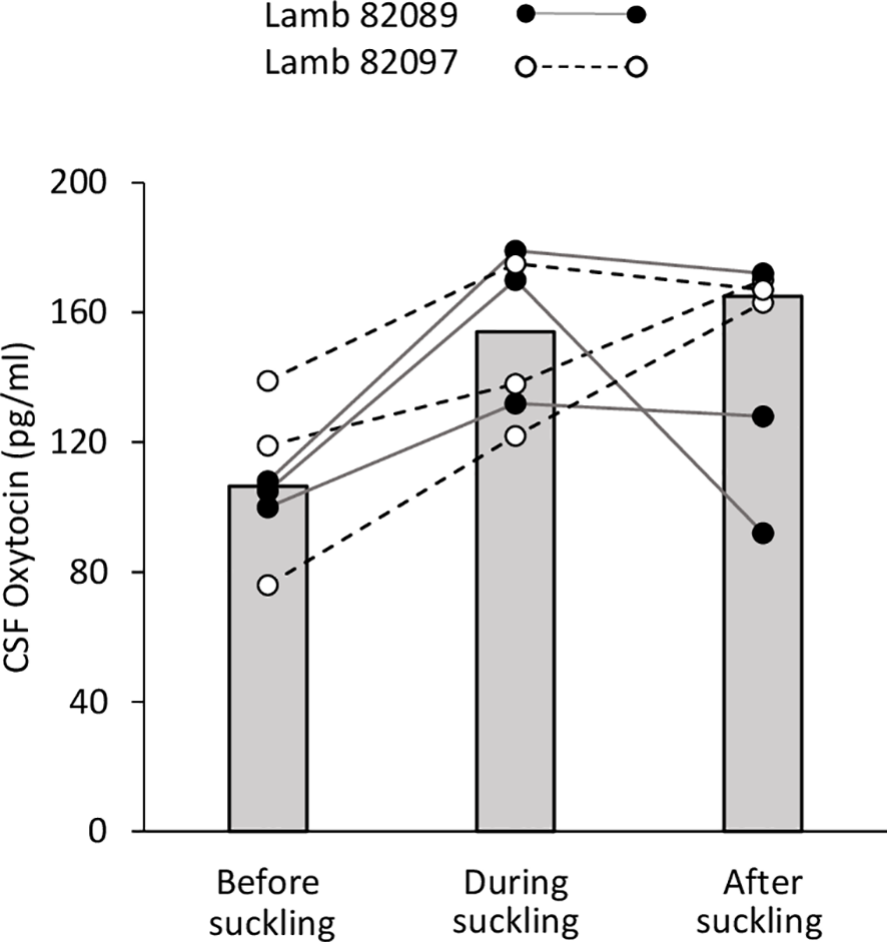
Effect of suckling on oxytocin levels in the CSF. Samples were taken during a 5-min period each time, before, during and after suckling from two lambs (82089 and 82097). Data are expressed as medians (gray bars) and individual data points.

Because lambs had been separated from their mothers at birth and were artificially fed, they developed strong affiliative relationships with the caregiver who provided milk daily. There was no difficulty in training the lambs to be restrained in the harness and taking CSF samples. As only two lambs were sampled in this preliminary trial, no statistical comparisons were made and individual data points are only described (**Figure 4**). Subjects were successfully sampled but the third value taken from lamb n°82097 was omitted because it was above the assay upper detection limit. The median value for all OT samples before bottle-feeding was 106.5 pg/ml (range: 76.0–139.0). For both lambs, all OT data points obtained in the CSF during the 5-min session of bottle-feeding were above those measured before (median value 153.9 pg/ml; range: 122.5–179.8). In the following minutes, the levels kept increasing or remained high in five samples, and decreased in one (median: 165.6 pg/ml; range: 92.9–173.0).

### Experiment 3. Early Infant Attachment Is Delayed by an OT-Receptor Antagonist

#### Behavior and Body Weight

**Figure 5:**
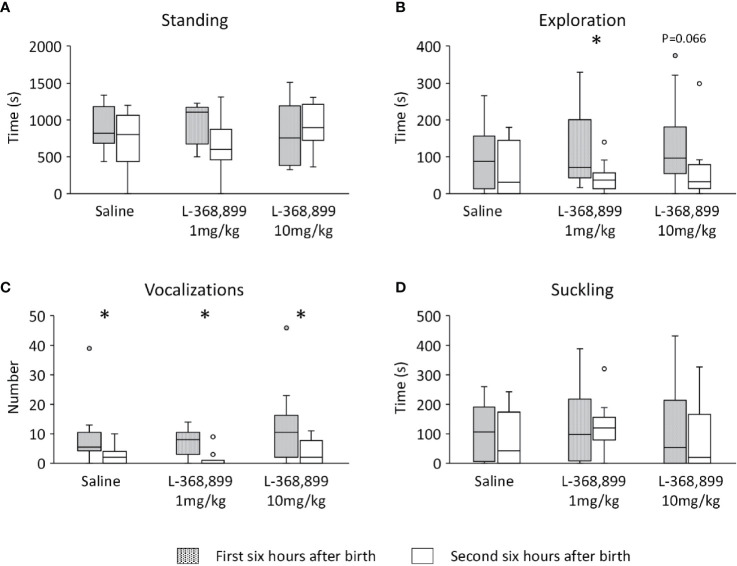
Lambs neonatal activities during the first 12 h after birth: **(A)** standing, **(B)** exploring the mother’s body (nosing, nuzzling), **(C)** number of vocalizations, and **(D)** suckling. Activities are represented in the first and second 6-h period of observation. At birth, lambs had unlimited access to the mother’s udder and received either saline (n=10) or L3688-899 at 1 mg (n=11) or 10 mg/kg (n=11) *via* orogastric tubing. Treatments were administered at birth, and then 2 and 4 h later. Data are expressed as medians and 1^st^ and 3^rd^ quartile. Paired box-plots associated with a star (*) are significantly different (p<0.05).

An analysis of the neonatal activities over time was inconclusive as these were extremely variable, some lambs being active during a specific observation session, while others were resting or sleeping. Therefore, to reduce the behavioral variability, the data were pooled in two time windows: the first 6 h when mothers and lambs were in individual pens, and the next 6 h when they were in communal pens with peers. No significant differences were found between treatments in the time spent standing (**Figure 5A**), but lambs receiving 1 mg/kg of L-368,899 explored less their mothers’ body once in the communal pen (Wilcoxon test: V=60, n=11, p=0.018, **Figure 5B**). A similar tendency was observed in those that received 10 mg/kg of L-368,899 (Wilcoxon test: V=46, n=10, p=0.066, **Figure 5B**). Lambs from the three treatments vocalized more in the first than in the second half of observations (Wilcoxon test: Controls: V=36, n=10, p=0.014; 1 mg/kg: V=36, n=11, p=0.014; 10 mg/kg: V=43.5, p=0.015; **Figure 5C**) and no significant differences were found in the suckling activity (**Figure 5D**).

Birthweight ranged from 2.2 to 5.6 kg and did not vary significantly between the three groups. Repeated measures ANOVA revealed a significant increase in body weight over time (F_(2,58)_=92.039, n= 32, p<0.0001) but no treatment*time interaction. Between birth and 48 h, the mean growth rate was 0.35 ± 0.17 kg and was not affected by the treatment.

#### Two-Choice Test

##### Preference for the Mother at 24 h

**Figure 6:**
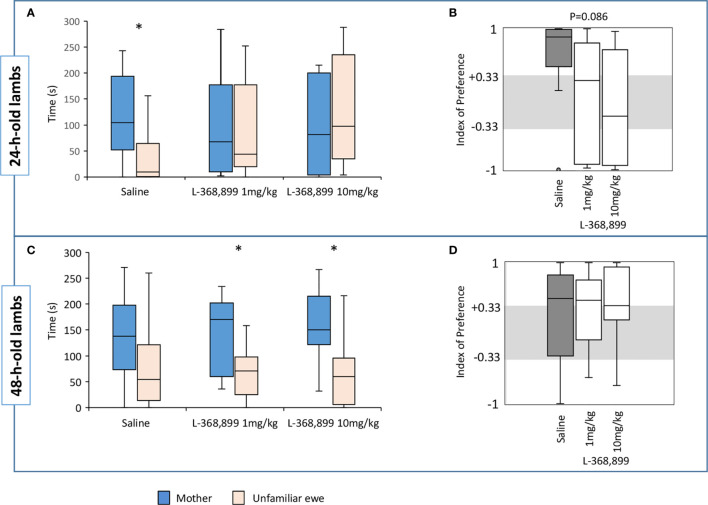
Time(s) spent in the contact zone near the mother or the unfamiliar ewe in the choice test by lambs when 24 h **(A)** and 48 h old **(C)**. At birth, lambs had unlimited access to the mother’s udder and received either saline (n=10) or L3688-899 at 1 mg (n=11) or 10 mg/kg (n=11) *via* orogastric tubing. Treatments were administered at birth, and then 2 and 4 h later. Indexes of preference (IP) in 24 h **(B)** and 48 h old lambs **(D)**. IP>+0.33: preference for the mother; +0.33<IP<-0.33: no preference; IP<-0.33: preference for the unfamiliar ewe. Data are expressed as medians and 1^st^ and 3^rd^ quartiles. Paired box-plots associated with a star (*) are significantly different (p<0.05).

We did not find any significant differences between the three groups in the latency to reach the zone of contact, nor in the total time spent in the contact zones or only next to the mother. On the other hand, a strong tendency was revealed in the time spent next to the unfamiliar ewe (Kruskal-Wallis test: K_obs_=5.94, df=2, n=10 to 11, p=0.051). Pairwise comparisons using Dunn’s procedure showed that Saline lambs spent less time near their unfamiliar ewe than those receiving 10 mg/kg of L-368,899 (n=10 in both groups, p=0.016; Bonferroni correction set at p=0.0167). Saline lambs also spent significantly more time near their mothers than near the unfamiliar ewes (Wilcoxon test: V=49, n=10, p=0.032) which was not the case for any of the lambs receiving the OT receptor antagonist (**Figure 6A**). In addition, a tendency for a difference was observed for the IP score (Kruskal-Wallis test: K_obs_=4.91, df=2, n=10 to 11, p=0.086; **Figure 6B**). Subsequent pairwise comparisons using Dunn’s procedure showed that the IP score was higher in lambs receiving saline than in those treated with 10 mg/kg of L-368,899, (n=10 and 11 respectively, p=0.029) but this was not considered statistically relevant after Bonferroni correction (set at p=0.0167). Nonetheless, most Saline lambs clearly preferred to stay next to their mothers being the only group with a median IP value above the threshold (+0.33): 8/10 of them choosing the mother, and only one spending all its time near the unfamiliar ewe. In those treated with L-368,899, the choice appeared random since the proportion of lambs having an IP above +0.33 or below -0.33 was rather similar (respectively: 5/11 vs. 4/11 for 1 mg/kg, and 4/11 vs. 5/11 for 10 mg/kg), the other lambs making no clear choices.

##### Preference for the Mother at 48 h

There was no reliable difference between groups in the latency to reach the zone of contact, neither in the total time spent in the contact zones, next to the mother or to the unfamiliar ewe, nor in the IP scores. Lambs that had received L-368,899 spent more time near their mothers than near the alien ewe (Wilcoxon test: 1 mg/kg: V=57, n=11, p=0.032; 10 mg/kg: V=58, n=11, p=0.024), while the difference did not reach statistical significance in Saline lambs (**Figure 6C**). However, median IP scores were above +0.33 in the three groups (**Figure 6D**) meaning that more than half the lambs clearly chose their own mothers. Individual values show that the proportion of lambs having an IP score above +0.33 or below -0.33 was rather equivalent in all groups (Saline: 6/10 vs. 1/10; 1 mg/kg: 7/11 vs. 1/11; 10 mg/kg: 7/11 vs. 1/11).

## Discussion

The development of a preference for the mother after birth is dependent on the success of the first suckling bouts during which colostrum, as an internal signal, plays a key role (30–32). This is an initial and crucial step leading to infant attachment in sheep (27). The present study shows that early suckling activity and OT are closely linked and that delaying suckling or using L-366,899, an antagonist that can cross the blood-brain barrier, affects the relationship with the mother. The outcome of the treatments are specific to the expression of affiliative behaviors and suggests that the oxytocinergic neural system, influencing a wide range of social behaviors, is fully active in newborn lambs and shapes the early development of infant attachment.

Preventing lambs from suckling for the first 6 h impairs the development of a preference for the mother in Experiment 1 and confirms previous results (30, 31). What is more unexpected is that shorter delays have also detrimental consequences. Allowing access to the udder 2 h after birth is not efficient for lambs to be able to discriminate their mothers from an unfamiliar ewe when 12 h old, and the extend of the consequences in the following day is directly linked to the duration of the delay. Only unrestricted access to the udder leads to proper expression of a preference for the mother within 12 h of life. The general activity of the lambs during the two-choice tests was not affected leading to the conclusion that only the abilities to display social preferences were altered. Individual IP values show that, regardless of the duration of the delay, the choice of mother or unfamiliar ewe is rather random at 12 h and also, to a lesser degree, at 24 h. Gradually, IP scores improved so that by 72 h of age, most lambs displayed a preference for their mothers. However, as we did not analyze the behavioral expression of the ewes, we cannot know to what extend it could have influenced the choice of the lamb. Both ewes were quite vocal in the test, calling for their lambs emitting mainly high-pitched and sometimes low-pitched bleats. Previous studies have shown that in the choice test, mothers display acceptance behavior in the presence of their own lamb while the other maternal ewe show rejection behavior and may be more vocal (59, 60). At 12–24 h, discrimination is based on such differential expression rather than of true recognition of maternal individual characteristics. By 48 h of age, identification of the mother’s voice based on acoustic structure is clearly established (61). We can only assume that the behavior of the ewes in the present study showed the same differential trend, in all experimental groups and at the three periods of test. Using multiparous ewes, accustomed to human presence and handling, made it very likely. The progressive development of early filial attachment results from learning processes (regardless of what is learned) in which suckling, and the comforting presence of the mother, motivate the lamb to maintain contact with the caregiver and learn her characteristics.

That only a few suckling episodes facilitate early learning of single sensory information had been shown at several occasions in mammals (dog pups: (62); rat pups: (63, 64); human babies: 65). The present work goes a step further by showing that suckling within 2 h after birth is the triggering factor that helps shaping early bonding with the mother. Observation of the neonatal activity showed that Control lambs got up onto four legs within half an hour after birth, and that they found the udder in the following 30 min. In any case, the slowest lamb managed to suckle 78 min after birth. Lambs were extremely active in the first 2 h of life, vocalizing, exploring the body of their mothers, and suckling, after which they alternated between resting and feeding. To be the most effective in terms of affiliative outcome, suckling must take place within a very narrow postnatal time window (less than 2 h). This suggests that the neonate must be in a specific neuroendocrinological state relating in all likelihood to the birth process. More than 30 years ago, Lagercrantz and Slotkin (66) hypothesized that the catecholamine surge due to vaginal birth was responsible for infant arousal and could facilitate attachment between mother and child. Since then, Varendi et al. (67) have shown that labour contractions enhance learning of natural breast odours in human neonates, and that noradrenalin may be responsible for it; the possible link between the neuroendocrinology of child birth and mother-infant attachment is now seriously taken into account (68). The same mechanisms may concern the development of a preference for the mother since lambs, like other mammals, experience a surge of catecholamine concentration during the perinatal period (69). Indeed one must not underestimate the fact that delayed suckling may have general metabolic effects that jeopardized neonatal activity and learning. For instance, lambs deprived of suckling for 6 h lost some weight and also had slightly lower rectal temperatures compared to unrestricted lambs, even though the data never revealed any sign of hypothermia. However, this must not be considered as alarming since losing weight around birth is usual in lambs even under normal circumstances (31, 37), a phenomenon that is the combined consequences of the coat drying out (due to maternal licking) and neonatal urination and defecation. In addition, while a delay in colostrum intake may impair general homeostasis, lambs deprived of suckling for a shorter time did not show any sign of weakness. Yet, at the age of 12 h they had poor discriminative abilities, and in some, this was still visible at the age of 24 h. It may be that the development of early infant attachment relies on the combined effects of two mechanisms: early suckling and gastric filing first, which must take place in the first 2 h, followed by the postprandial neuroendocrine consequences of colostrum ingestion that reinforce the initial step. This would explain why the impact on affiliative behaviors is directly linked to the duration of the delay in the first suckling episode. Beyond a short postnatal time window, subsequent feeding bouts cannot compensate for the lack of successful suckling at birth. Significant deleterious effects can be seen 24 h later in lambs that did not have access to the udder in the 4 to 6 h of life, a result that confirms previous studies (30, 31).

Our study also reveals that suckling triggers the release of OT in the plasma and this may also be the case in the cerebrospinal fluid. Findings from Experiment 2 show that OT levels increase during feeding and, like in the calf (44), it is very rapid and short-lasting. This suggests that somato-sensory stimulation from the orogastric sphere (sucking, swallowing, gastric distension) may stimulate the release of OT. It is known that gastric distension induced by infusion of mother’s milk or saline triggers a rise of OT in 10-day-old rats (70). In addition, OT levels may also be elevated through the effect of endocrine factors such as the secretion of the gastrointestinal hormone CCK. This could well be the case in the lamb since CCK is released after suckling (30, 33) and it is known, in the rat, that both CCK and gastric distension activate the oxytocinergic system in the hypothalamus, and trigger the release of OT in the plasma (71, 72). Preliminary data from Experiment 2 demonstrate that OT concentrations also rise in the cerebrospinal fluid after bottle-feeding, and that the phenomenon is quite repeatable. While we cannot draw definite conclusions from only two subjects, one can assume that the first suckling bouts at birth lead to the same outcome. This is supported by a previous study showing that orogastric infusions of colostrum or saline in newly born lambs activate hypothalamic nuclei producing OT as well as vasopressin (73). Our present results did not show any change in plasma OT concentrations when close contacts excluded suckling. This was rather unexpected since Guesdon et al. (45) have revealed that in a context where lambs can develop a bond with a human being, social interactions with the caregiver activates the oxytocinergic neurons of the hypothalamus. It may be that our experimental situation was not suitable to detect a peripheral response. Or else, non-nutritive interactions may trigger a response in the brain without any visible release in the blood. There is evidence in humans that saliva is a better medium than plasma to reveal changes in the cerebrospinal fluid (74). Even though salivary OT is suspected to emanate from local production, it may be functionally linked to the central nervous system (75). This link could explain why salivary OT levels have been shown to increase in 4–6 months infants when engaging in “play-and-touch” with their parents (43), and in 7–12 years daughters when being comforted by their mothers (76). However, drawing clear-cut conclusions on OT plasma levels relating to touch is not easy. Studies in humans often show that ELISA reveals clearer patterns that RIA, suggesting that diverse molecular structures, linear or shorter forms, or even metabolites may be detected by the former method (75, 77, 78). Our ELISA method showing that suckling but not close social interactions releases OT in the plasma is more congruent with studies using RIA in humans and raises questions. One observation to bear in mind, on the other hand, is that our experimental situation imposed a constraint: when the lambs were reunited with their mothers after overnight separation, suckling was not made possible since the udder was covered. This could have been frustrating since the release of OT in response to social interaction is usually revealed upon natural and voluntary contact, and is seen as the expression of positive emotions (39, 79, 80). It may be why Guesdon et al. (45) showed by immunohistochemistry that OT neurons were activated when hand-reared lambs interact socially and voluntarily with their human caregivers. Stroking them gently does not enhance the response. That OT is not released following social touch in the lamb cannot be a firm and definite conclusion. Finally, our results show that, despite similar profiles, differences in plasma levels are more contrasted in the third than in the first session. In the third session, OT plasma concentrations obtained immediately after 3 min of suckling are significantly higher than at any other time point which is not the case in the first session. This reflects a lower variability in the physiological response as lambs became accustomed to the handling procedure.

In our last experiment, we showed that orogastric administration of the OT receptor antagonist L-368,899 altered the onset of early infant attachment. The major outcome is the impaired discriminative abilities in 24-h-old lambs, and both doses were as efficient. One important fact is that the effects were only visible on affiliative behaviors. During the choice test, the general activity of the lambs was in no way affected: le latency to reach the ewes and the time spent in the contact zones did not differ between treatments. By contrast, while lambs receiving saline showed very good discriminative abilities (8/10 clearly sought contact with the mother), lambs treated with the OT receptor antagonist chose a ewe randomly. The same comment as in Experiment 1 can be made regarding the behavioral expression of the ewes that we did not take into account. Previous work dealing with a CCK receptor antagonist led to the same impairment in lambs’ discriminative abilities, despite the fact that mothers unlike the other maternal ewes had displayed acceptance behavior (37). We assume that this was also the case in Experiment 3. It seems that repressing OT activity modifies specifically the expression of infant social behavior. Interestingly, while observation of the lambs’ activity over the first 12 h after birth did not reveal any significant differences between treatments, the exploratory behavior pattern was not quite the same. Exploration consists in lambs nosing and sniffing their mothers’ body and reflects close social interactions. It decreased in lambs treated with L-368,899 when they were transferred from the individual to the communal pen with their mothers. This was not the case for Saline lambs suggesting that the OT receptor antagonist may have altered the ability of lambs to remain close to their mothers and engage in prosocial behaviors. There is evidence in other species of the involvement of OT in triggering close social contact or grooming [rat: (81); marmoset: (56); naked mole-rat: (57); common vampire bat: (82)], the same could apply to proximal interactions between the lamb and its mother. However, the effect was only revealed in a challenging situation, when several lambs and ewes were housed together. This context imposes more constraints on mothers and young to stay together since there is a risk of mixing or separating progenies, which is not the case in individual pens.

Results from these three experiments are closely related: (i) suckling triggers the release of OT in the plasma and in the cerebrospinal fluid, (ii) depriving lambs from suckling immediately at birth affects the development of a preference for the mother, (iii) as does the use of the OT receptor antagonist L-368,899. It is therefore tempting to conclude that it is through the activation of the oxytocinergic system that neonatal suckling favours the development of infant attachment. One limitation of the present work is that we cannot conclude with certainty that L-368,899 acted on central OT. There is evidence that this antagonist enters rapidly the blood stream in the dog with a half-life of 1.7 to 2.7 h (54). It is detected in the cerebrospinal fluid of Rhesus 40 min after injection and still present 2 h later, accumulates in the hypothalamus and limbic structures identified as containing OT receptors, and induces a lack of interest for infants in adults (55). L-368,899 has been shown to alter prosocial behaviors in other species [Rhesus macaque: (55); marmoset: (56); naked mole-rat: (57); prairie vole: (83)] and in many cases authors also validated their results with the complementary use of OT. That OT plays a major role in affiliative behaviors between parents and infants is now widely accepted (39–41, 75, 78, 84, 85). Despite our concern, there is good reasons to believe that this could be the case in the lamb as well.

Sheep are known to develop a strong mother-young bond based on mutual recognition that takes place rapidly around parturition (6, 26, 85). The importance of OT in the onset of maternal behavior in the ewe has been clearly demonstrated (84). Kendrick (84) has also shown that ewes given intracerebroventricular infusions of OT bond selectively with their lambs, while OT antagonist prevents it. The current study shows that L-368,899 alters the development of a bond with the mother. In the light of these results, it appears that the sheep is actually the only animal species providing experimental evidence that mutual mother-infant attachment and OT are closely related.

## Data Availability Statement

The raw data supporting the conclusions of this article will be made available by the authors, without undue reservation.

## Ethics Statement

Animal requirements were in accordance with the European Community Council Directive 2010/63/UE. The experimental facilities had received authorization to house experimental animals from the local bureau of veterinary services (Indre-et-Loire, France, authorization N°: E 37-175-2) and all the experimental procedures were approved by the Val de Loire Ethic Committee (authorization N°2012-08-11). All the personnel involved had special training in animal care, handling and experimentation required by the French Ministry of Agriculture. Except for the two cannulated lambs, ewes and lambs returned to the breeding research unit at the end of each experiment.

## Author Contributions

RN, MK, FL, and EC designed the study and contributed significantly in the writing of the manuscript. FC, RN, MK, FL, and EC collected data. RN, MK, and EC analyzed the data. JC and FC performed neurosurgery. PW provided generously L-368,899. P-GM performed OT assays. RN was in charge of Experiments 1 and 2 and MK of Experiment 3. RN was chief investigator of the research program ANR BOND.007. All authors contributed to the article and approved the submitted version.

## Funding

This work was funded by the Agence Nationale de la Recherche (ANR BOND.007, no. NT09 492885) and was made possible through support from the GDR2822 ‘‘Ethologie’’.

## Conflict of Interest

The authors declare that the research was conducted in the absence of any commercial or financial relationships that could be construed as a potential conflict of interest.
